# Food insecurity and physical multimorbidity among adults aged ≥ 50 years from six low- and middle-income countries

**DOI:** 10.1007/s00394-022-02999-5

**Published:** 2022-09-21

**Authors:** Lee Smith, Jae Il Shin, Louis Jacob, Guillermo F. López Sánchez, Felipe Schuch, Mark A. Tully, Hans Oh, Nicola Veronese, Pinar Soysal, Laurie Butler, Yvonne Barnett, Ai Koyanagi

**Affiliations:** 1 Centre for Health, Performance and Wellbeing, Anglia Ruskin University, Cambridge, UK; 2 Department of Pediatrics, Yonsei University College of Medicine, Seoul, 03372 Korea; 3 Research and Development Unit, Parc Sanitari Sant Joan de Déu, CIBERSAM, ISCIII, Dr. Antoni Pujadas, 42, Sant Boi de Llobregat, 08830 Barcelona, Barcelona Spain; 4 Faculty of Medicine, University of Versailles Saint-Quentin-en-Yvelines, 78000 Versailles, France; 5 Division of Preventive Medicine and Public Health, Department of Public Health Sciences, School of Medicine, University of Murcia, Murcia, Spain; 6 Department of Sports Methods and Techniques, Federal University of Santa Maria, Santa Maria, Brazil; 7 School of Health Sciences, Institute of Mental Health Sciences, Ulster University, Newtownabbey, BT15 1ED Northern Ireland; 8 Suzanne Dworak Peck School of Social Work, University of Southern California, Los Angeles, CA 90007 USA; 9 Department of Internal Medicine and Geriatrics, University of Palermo, 90133 Palermo, Italy; 10 Department of Geriatric Medicine, Bezmialem Vakif University, 34093 Istanbul, Turkey; 11 ICREA, Pg, Lluis Companys 23, 08010 Barcelona, Spain

**Keywords:** Multimorbidity, Food insecurity, Chronic disease, Low- and middle-income countries, Older adults

## Abstract

**Purpose:**

Food insecurity and multimoribidity (i.e., ≥ 2 chronic conditions) may be linked bidirectionally, but there are no studies on this topic from LMICs. Therefore, the aim of the present study was to examine the association between food insecurity and physical multimorbidity in a large representative sample of older adults from six LMICs.

**Methods:**

Cross-sectional, community-based data on adults aged ≥ 50 years from the World Health Organization’s Study on Global AGEing and Adult Health (SAGE) conducted in China, Ghana, India, Mexico, Russia, and South Africa were analyzed. A total of 11 chronic physical conditions were assessed. Past 12 month food insecurity was assessed with two questions on frequency of eating less and hunger due to lack of food. Multivariable logistic regression analysis was conducted to assess the associations.

**Results:**

Data on 34,129 adults aged ≥ 50 years [mean (SD) age 62.4 (16.0) years; age range 50–114 years; 47.9% males] were analyzed. After adjustment for potential confounders, in the overall sample, compared to being food secure, moderate and severe food insecurity were associated with 1.29 (95% CI 1.06–1.56) and 1.56 (95% CI 1.13–2.16) times higher odds for multimorbidity, respectively

**Conclusion:**

Food insecurity was associated with greater odds for multimorbidity in older adults from LMICs. Addressing food insecurity in the general population may reduce risk for multimorbidity, while screening for food insecurity and addressing it among those with multimorbidity may lead to better clinical outcomes, pending future longitudinal research

**Supplementary Information:**

The online version contains supplementary material available at 10.1007/s00394-022-02999-5.

## Introduction

Multimorbidity is often defined as “the coexistence of two or more chronic diseases” in the same individual [1]. Multimorbidity is a growing global public health challenge, as populations age and the prevalence of long-term conditions rise [2, 3]. This is of concern as multimorbidity is associated with poorer health outcomes (e.g., functional limitations, falls, premature mortality) and increased use of health and social care services with associated costs [4–8]. Such burden may be most pronounced in low- and middle-income countries (LMICs) where 80% of chronic diseases occur [9], and where the health and social care systems are under resourced, and perform poorly in comparison to high-income countries (HICs) [10]. It is thus important to identify correlates of multimorbidity in LMICs to inform targeted interventions.

One potential correlate of multimorbidity that has been little studied to date is food insecurity. Food insecurity may be defined as the disruption of food intake or eating patterns due to lack of money and other resources [11], and is most pronounced in LMICs [12–14]. Malnutrition, as a result of food insecurity, is strongly associated with vitamin deficiency, and in turn, this is associated with multiple chronic diseases such as osteoporosis, osteomalacia, and cardiovascular disease [15, 16]. Furthermore, when food becomes scarce, people tend to shift to more affordable but less nutritious food (e.g., high fat and carbohydrates), and this can also lead to an increased risk for cardiovascular diseases, for example [17]. Finally, it is also possible that multimorbidity leads to an increased risk for food insecurity, possibly due to financial burden as a consequence of suffering from multiple conditions [18, 19]. This implies a vicious cycle where people experiencing food insecurity may be at increased risk of multimorbidity due to poor nutrition, and multimorbidity may further exacerbate food insecurity via impoverishment. This vicious cycle may be particularly pronounced in many LMICs where universal health coverage is scarce, and where out-of-pocket expenditure and even catastrophic health expenditure are common.

However, to date, there is a scarcity of research on the association between food insecurity and multimorbidity. In one cross-sectional study of 2048 US older adults, compared to those without multimorbidity (i.e., 0–1 chronic conditions), food insecurity was positively associated with multiple chronic conditions (RRR 1.60, 95% CI 1.08–2.36, for 2–3 conditions; RRR 2.59, 95% CI 1.55–4.33 for 4–10 chronic conditions) [20]. Another cross-sectional study found that food insecurity was associated with greater than three times higher odds of multimorbidity in a sample of older US women (*n* = 279) receiving home-delivered meals [21]. Two other cross-sectional studies from the US and the US and Canada found that those with multimorbidity are at an increased risk of food insecurity [18, 19]. Specifically, in the study carried out in the US (*n* = 3552), compared to those with 0–1 conditions, respondents with multiple chronic conditions were significantly more likely to report food insecurity, with the adjusted odds ratio (OR) for those with 2–4 conditions being 2.12 (95% CI 1.45–3.09) and for those with five or more conditions being 3.64 (95% CI 2.47–5.37) [18]. In the study carried out in the US and Canada (*n* = 77,053), compared with adults with no chronic conditions, the odds for household food insecurity were 1.43 (95% CI 1.28–1.59), 1.86 (95% CI 1.62–2.14), and 3.44 (95% CI 3.02–3.93) times higher among adults with 1, 2, and 3 or more chronic conditions, respectively. To date, to the best of our knowledge, no other study exists on this topic.

The existing literature has some clear limitations. First, all studies were carried out in HICs (US and Canada), and the magnitude of this association in LMICs, where the prevalence of food insecurity is high, and where health services can be fragmented, under resourced and underperform, is not known [10]. Furthermore, disease profiles in LMICs are often different from those of high-income countries, and thus, findings from HICs are unlikely to be generalizable to LMICs. Finally, all existing studies have focused on one or two HICs. Multi-country studies from a variety of settings are important as they allow for the comparison of standardized estimates across settings. Given this background, the aim of the present study was to examine the cross-sectional association between food insecurity and physical multimorbidity in a sample of 34,129 adults aged ≥ 50 years from six LMICs spanning multiple continents. We hypothesized that food insecurity will be associated with higher odds for physical multimorbidity in LMICs.

## Methods

Data from the Study on Global Ageing and Adult Health (SAGE) were analyzed. These data are publically available through https://apps.who.int/healthinfo/systems/surveydata/index.php/catalog/sage/about

This survey was undertaken in China, Ghana, India, Mexico, Russia, and South Africa between 2007 and 2010. Based on the World Bank classification at the time of the survey, Ghana was the only low-income country, and China and India were lower middle-income countries although China became an upper middle-income country in 2010. The remaining countries were upper middle-income countries. Details of the survey methodology have been published elsewhere [22]. Briefly, in order to obtain nationally representative samples, a multistage clustered sampling design method was used. The sample consisted of adults aged ≥ 18 years with oversampling of those aged ≥ 50 years. Trained interviewers conducted face-to-face interviews using a standard questionnaire. Standard translation procedures were undertaken to ensure comparability between countries. Half of the interviews in China were completed using a computer-assisted personal interview (CAPI) and the other half used paper and pencil. Mexico used CAPI and the other four countries used paper and pencil format for all interviews. The survey response rates were: China 93%; Ghana 81%; India 68%; Mexico 53%; Russia 83%; and South Africa 75%. Sampling weights were constructed to adjust for the population structure as reported by the United Nations Statistical Division. Ethical approval was obtained from the WHO Ethical Review Committee and local ethics research review boards (ethical approval numbers not available). Written informed consent was obtained from all participants.

### Chronic physical conditions and physical multimorbidity

We included all 11 chronic physical conditions for which data were available in the SAGE. *Chronic back pain* was defined as having had back pain everyday during the last 30 days. Respondents who answered affirmatively to the question “Have you lost all of your natural teeth?” were considered to have edentulism. The participant was considered to have hearing problems if the interviewer observed this condition during the survey. Hypertension was defined as having at least one of the following: systolic blood pressure ≥ 140 mmHg; diastolic blood pressure ≥ 90 mmHg; or self-reported diagnosis. Visual impairment was defined as having severe/extreme difficulty in seeing and recognizing a person that the participant knows across the road [23]. Diabetes and stroke were solely based on lifetime self-reported diagnosis. For other conditions, the participant was considered to have the condition in the presence of either one of the following: self-reported diagnosis; or symptom-based diagnosis based on algorithms. We used these algorithms, which have been used in previous studies using the same dataset, to detect undiagnosed cases [24, 25]. Specifically, the validated Rose questionnaire was used for angina [26], and other previously validated symptom-based algorithms were used for arthritis, asthma, and chronic lung disease [24]. Further details on the definition of chronic physical conditions can be found in Table S1 (Appendix). The total number of chronic physical conditions was calculated and multimorbidity was defined as ≥ 2 chronic physical conditions, in line with previously used definitions [25].

### Food insecurity

Food insecurity was defined by the two following questions: “In the last 12 months, how often did you ever eat less than you felt you should because there wasn’t enough food?” and “In the last 12 months, were you ever hungry, but didn’t eat because you couldn’t afford enough food?” Both of these questions had as response options: every month (coded = 1); almost every month (coded = 2); some months, but not every month (coded = 3); only in one or 2 months (coded = 4); never (coded = 5). These items were based on similar items found in food security questionnaires such as the US Household Food Security Survey Module and National Health and Nutrition Examination Survey (NHANES) Food Security module. As in a previous SAGE study, those who answered one through three to both questions or answered one to either item were categorized as severely food insecure. Those who did not fulfill the criteria for severe food insecurity but answered two through four for either question were coded as moderately food insecure. Those who answered five to both items were categorized as food secure [27]. We also used the dichotomous variable of any food insecurity (i.e., moderate/severe or none) in some analyses.

### Control variables

The selection of control variables was based on previous literature [28] and included age, sex, highest level of education achieved (≤ primary, secondary, tertiary), and wealth quintiles based on income.

### Statistical analysis

The statistical analysis was done with Stata 14.2 (Stata Corp LP, College station, Texas). The analysis was restricted to those aged ≥ 50 years. Tetrachoric correlations were assessed between each pair of chronic conditions among those with any food insecurity (*N* = 5444). Using the overall sample, multivariable logistic regression analyses were conducted to assess the association of food insecurity level (i.e., food secure, moderate, severe) or any food insecurity (exposure variables) with individual chronic conditions or multimorbidity (i.e., ≥ 2 chronic conditions) as the outcomes. The analysis on food insecurity and multimorbidity was also stratified by country. All regression analyses were adjusted for age, sex, education, wealth, and country, with the exception of the country-wise analysis which was not adjusted for country. Adjustment for country was done by including dummy variables for each country in the model as in previous SAGE publications. The sample weighting and the complex study design were taken into account in all analyses. Results from the regression analyses are presented as odds ratios (ORs) with 95% confidence intervals (CIs). The level of statistical significance was set at *P* < 0.05.

## Results

**Fig. 1:**
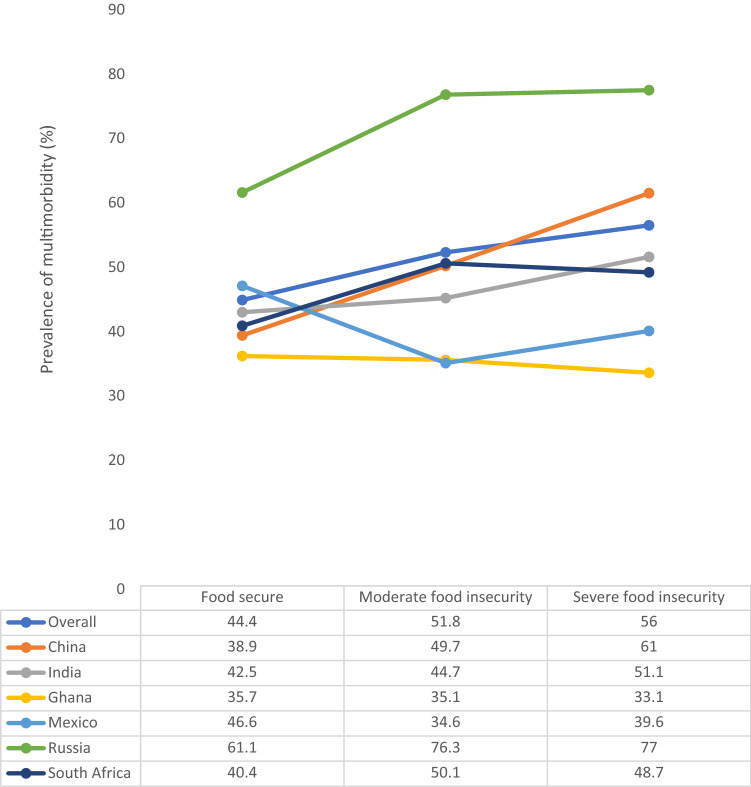
Prevalence of physical multimorbidity by food insecurity status (overall and by country)Multimorbidity referred to ≥ 2 chronic conditions

The final sample consisted of 34,129 adults aged ≥ 50 years [mean (SD) age 62.4 (16.0) years; age range 50–114 years; 47.9% males]. The sample characteristics are provided in Table 1. Overall, the prevalence of multimorbidity was 45.5%, while 6.7 and 5.1% had moderate and severe food insecurity, respectively. The most common chronic condition was hypertension (55.0%), followed by arthritis (29.5%), and angina (17.6%). The strongest pairwise correlations among those with any food insecurity was observed for chronic lung disease and angina or asthma (Table 2). Overall, the prevalence of multimorbidity increased with increasing severity of food insecurity (Fig. 1). However, not all countries showed similar trends. For example, increasing trends were not shown in Ghana and Mexico. After adjustment for potential confounders, any food insecurity was significantly associated with higher odds for arthritis, asthma, chronic lung disease, hearing problems, and visual impairment (Table 3). Compared to moderate food insecurity, severe food insecurity was more strongly associated with all these conditions. In the overall sample, compared to being food secure, moderate and severe food insecurity were associated with 1.29 (95% CI 1.06–1.56) and 1.56 (95% CI 1.13–2.16) times higher odds for multimorbidity, respectively (Table 4). Any food insecurity was significantly associated with multimorbidity in China, India, Russia, and South Africa but not in Ghana and Mexico.

**Table 1:** Sample characteristics (overall and by country)

Characteristic	Total *N* = 34,129	China*N* = 13,175	Ghana *N* = 4305	India *N* = 6560	Mexico *N* = 2313	Russia *N* = 3938	South Africa *N* = 3838
Multimorbidity							
Yes	45.5	39.1	35.1	43.4	43.4	63.3	43.1
Food insecurity level							
Food secure	88.2	98.8	55.4	81.5	64.9	86.1	67.6
Moderate	6.7	0.9	23.5	11.1	15.3	7.7	10.9
Severe	5.1	0.3	21.0	7.5	19.7	6.2	21.5
Age (years)							
Mean (SD)	62.4 (16.0)	62.6 (16.7)	61.5 (13.7)	64.4 (19.9)	63.0 (18.9)	63.9 (15.4)	61.6 (18.4)
Sex							
Male	47.9	49.8	52.4	51.0	46.8	38.9	44.1
Education							
≤ Primary	57.4	63.0	75.3	76.1	79.6	7.5	71.4
Secondary	35.2	32.5	21.1	18.8	12.3	74.2	22.8
Tertiary	7.4	4.5	3.6	5.1	8.1	18.2	5.7
Angina							
Yes	17.6	9.4	12.8	17.0	6.7	37.3	8.9
Arthritis							
Yes	29.5	26.7	26.2	27.9	14.5	38.2	30.6
Asthma							
Yes	7.9	4.3	5.0	12.5	4.9	6.5	7.7
Chronic back pain							
Yes	8.6	5.6	7.5	9.6	8.4	13.0	5.7
Chronic lung disease							
Yes	15.8	11.3	3.7	17.2	13.2	24.4	7.4
Diabetes							
Yes	6.8	6.6	3.8	6.9	17.6	7.0	9.2
Edentulism							
Yes	12.9	9.1	3.0	15.1	21.7	18.1	8.5
Hearing problems							
Yes	5.6	5.5	2.9	5.6	9.3	6.1	5.0
Hypertension							
Yes	55.0	60.6	59.6	37.5	61.9	72.1	78.3
Stroke							
Yes	3.0	3.0	2.8	2.0	4.3	4.8	4.0
Visual impairment							
Yes	1.3	0.5	1.0	2.4	0.8	0.9	0.8

Data are % unless otherwise stated

*SD* standard deviation

**Table 2:** Tetrachoric correlations between each pair of chronic conditions among individuals with any food insecurity

	Angina	Arthritis	Asthma	Chronic back pain	Chronic lung disease	Diabetes	Edentulism	Hearing problems	Hypertension	Stroke	Visual impairment
Angina	1										
Arthritis	0.2679^a^	1									
Asthma	0.3729^a^	0.1822^a^	1								
Chronic back pain	0.3530^a^	0.3119^a^	0.2333^a^	1							
Chronic lung disease	0.5133^a^	0.2501^a^	0.6742^a^	0.3376^a^	1						
Diabetes	0.2135^a^	0.1421^a^	0.1282^a^	0.1384^a^	0.2400^a^	1					
Edentulism	0.1480^a^	0.1103^a^	0.1054^a^	0.1605^a^	0.2878^a^	0.1943^a^	1				
Hearing problems	0.1277^a^	0.1290^a^	0.1009^a^	0.1120^a^	0.1984^a^	0.1148^a^	0.1810^a^	1			
Hypertension	0.0782^a^	0.1279^a^	0.0064	0.039	0.0247	0.2597^a^	0.0352	0.0943^a^	1		
Stroke	0.1793^a^	0.2365^a^	0.1700^a^	0.1562^a^	0.2275^a^	0.2794^a^	0.0991	0.1844^a^	0.3261^a^	1	
Visual impairment	0.1815^a^	0.1425^a^	0.2103^a^	0.2635^a^	0.1646^a^	0.0917	0.0927	0.2307^a^	−0.0036	0.073	1

^a^*P* < 0.05

**Table 3:** Association between food insecurity and individual chronic conditions (outcomes) estimated by multivariable logistic regression

	Angina	Arthritis	Asthma	Chronic back pain	Chronic lung disease	Diabetes	Edentulism	Hearing problems	Hypertension	Stroke	Visual impairment
Food insecurity											
Food secure	1.00	1.00	1.00	1.00	1.00	1.00	1.00	1.00	1.00	1.00	1.00
Level											
Moderate	1.34 [0.98–1.81]	1.24^b^ [1.01–1.52]	1.21 [0.95–1.54]	1.14 [0.75,1.73]	1.51^d^ [1.21–1.87]	0.81 [0.60–1.10]	0.93 [0.68–1.27]	1.35 [0.94–1.93]	0.84 [0.71–1.00]	1.04 [0.58–1.86]	2.09^b^ [1.18–3.71]
Severe	1.09 [0.71–1.68]	1.70^c^ [1.17–2.46]	1.30 [0.98–1.73]	1.13 [0.79,1.62]	1.64^c^ [1.16–2.31]	0.85 [0.52–1.38]	0.92 [0.68–1.25]	1.53^b^ [1.06–2.20]	1.03 [0.78–1.35]	1.62 [0.97–2.69]	2.62^c^ [1.38–4.99]
Any food											
No	1.00	1.00	1.00	1.00	1.00	1.00	1.00	1.00	1.00	1.00	1.00
Insecurity^a^											
Yes	1.23 [0.92–1.65]	1.42^c^ [1.14–1.77]	1.25^b^ [1.03–1.52]	1.13 [0.83–1.55]	1.56^d^ [1.26–1.94]	0.83 [0.62–1.11]	0.93 [0.73–1.18]	1.42^b^ [1.09–1.86]	0.92 [0.78–1.08]	1.29 [0.88–1.88]	2.30^d^ [1.44–3.69]

Data are odds ratio [95% confidence interval]. Models are adjusted for age, sex, education, wealth, and country.

^a^“No” corresponds to food secure and “Yes” refers to moderate or severe food insecurity.

^b^*p* < 0.05, ^c^*p* < 0.01, ^d^*p* < 0.001

**Table 4:** Association between food insecurity and multimorbidity (outcome) estimated by multivariable logistic regression (overall and by country)

	Overall	China	India	Ghana	Mexico	Russia	South Africa
Food insecurity							
Food secure	1.00	1.00	1.00	1.00	1.00	1.00	1.00
Level							
Moderate	1.29^b^ [1.06–1.56]	1.68^b^ [1.03–2.75]	1.11 [0.88–1.39]	1.01 [0.81–1.25]	0.67 [0.37–1.19]	2.13^c^ [1.29–3.49]	1.57 [1.00–2.48]
Severe	1.56^c^ [1.13–2.16]	2.46^b^ [1.24–4.89]	1.46^b^ [1.07–2.00]	0.94 [0.76–1.17]	0.83 [0.47–1.49]	2.22 [0.66–7.55]	1.49^b^ [1.08–2.07]
Any food							
No	1.00	1.00	1.00	1.00	1.00	1.00	1.00
Insecurity^a^							
Yes	1.40^d^ [1.15–1.70]	1.84^c^ [1.22–2.77]	1.24^b^ [1.01–1.51]	0.98 [0.82–1.16]	0.76 [0.50–1.14]	2.17^b^ [1.13–4.15]	1.52^c^ [1.16–1.99]

Data are odds ratio [95% confidence interval]. Multimorbidity refers to ≥ 2 chronic conditions. Models are adjusted for age, sex, education, and wealth. Overall estimate is additionally adjusted for country.

^a^“No” corresponds to food secure and “Yes” refers to moderate or severe food insecurity.

^b^*p* < 0.05, ^c^*p* < 0.01, ^d^*p* < 0.001

## Discussion

### Main findings

In this large nationally representative sample of 34,129 adults aged ≥ 50 years, we found that compared to being food secure, moderate and severe food insecurity were associated with 1.29 (95% CI 1.06–1.56) and 1.56 (95% CI 1.13–2.16) times higher odds for multimorbidity, respectively. In terms of individual chronic conditions, any food insecurity was significantly associated with higher odds for arthritis, asthma, chronic lung disease, hearing problems, and visual impairment. However, country-wise analysis showed that food insecurity may not be associated with multimorbidity in all settings. Specifically, no significant associations were found in Mexico and Ghana.

### Interpretation of the findings

The findings of our study are broadly in line with previous studies on this topic from HICs, which have found a positive association between food insecurity and multimorbidity [20, 21]. There are several plausible pathways that likely explain the relationship between food insecurity and multimorbidity. First, food insecurity is associated with vitamin and mineral deficiency, and this may lead to the development of multiple chronic conditions via several biophysiological mechanisms (e.g., increased levels of inflammation) [29, 30].

It is also important to note that vitamin deficiency exacerbates chronic diseases [30], potentially leading to greater severity and an increased likelihood of diagnosis. For example, in the case of asthma, an unhealthy diet resulting from household food insecurity may influence systemic inflammation that contributes to asthma. Moreover, low levels of fruit and vegetable consumption, and the resultant reduction in antioxidants might reduce airway inflammation [31]. Second, food insecurity increases levels of chronic stress, and stress can increase visceral fat accumulation and risk for chronic diseases. Specifically, under severe stress conditions, both the hypothalamus–pituitary–adrenal axis and reward pathways can contribute to the release of cortisol, neuropeptide Y, and other substances, causing a desire to consume high energy-dense foods, and altered metabolism [32]. In turn, this may increase risk for chronic diseases, in particular, cardiovascular diseases. Apart from these biological mechanisms, this shift to poor diets in food insecurity can also be related with financial reasons (i.e., low-cost high energy foods) [33] and may increase risk for overconsumption of energy and resultant obesity, which is a risk factor of a variety of non-communicable diseases [33]. Furthermore, food insecurity may exacerbate existing health conditions for older adults due to financial challenges in decisions between purchasing food versus medication that can subsequently lead to poorer disease management, greater subsequent health care needs, and worse overall health [34].

On the other hand, it is also possible that having chronic health conditions can remove one from the labor market, and without stable income, this may result in food insecurity. Further, having multiple chronic health conditions may also be expensive (e.g., treatments, medications, caretakers) especially without reliable health insurance, causing an economic burden on individuals and families, which includes food insecurity. Finally, multimorbidity when accompanied by disability can limit self-efficacy, social interactions, and social functioning, and this may increase risk for food insecurity [35]. Therefore, it is likely that the relationship between food insecurity and multimorbidity is bi-directional.

In our study, significant associations between food insecurity and multimorbidity was not found in Mexico and Ghana, suggesting that this association may be context specific. Although the reasons for the non-significance in these two countries can only be speculated, this may be due to difference in quality of health care, universal health coverage, availability of low-cost high energy food, or multimorbidity patterns [25] that may differ between countries. For example, Mexico had a high prevalence of diabetes and edentulism, which were not associated with food insecurity. Alternatively, it is possible for people with multimorbidity to be more health conscious following the advice of doctors, and this may lead to an increased effort to avoid food insecurity in people with multimorbidity in some settings. However, clearly, further studies are necessary on this issue to clarify why food insecurity is not associated with multimorbidity in some settings.

### Public health implications

Findings from the present study suggest that addressing food insecurity in LMICs may lead to reduction in multimorbidity, or that among those with multimorbidity, addressing food insecurity may be important to improve clinical outcomes. Foodbanks have been found to provide immediate solutions to severe food deprivation and have the potential to improve food security outcomes in HICs [36]. In LMICs, such as African countries, the food bank model is still developing [37]. It may be prudent for governments and aid organizations to focus time and resource on developing the food bank model in LMICs to address food insecurity, which may have the additional benefit of reducing multimorbidity at a population level. Screening for food insecurity and addressing it among patients with multimorbidity in the clinical scene may also be an effective targeted strategy that may also improve clinical outcomes in these patients. For example, treatment may become incomplete in people who are food insecure for giving financial priority to food acquisition than treatment.

### Strengths and limitations

The large sample size consisting of multiple nationally representative surveys from LMICs, where no data on food insecurity and multimorbidity exist, are clear strengths of the study. Furthermore, our results are important given the current COVID-19 pandemic, where food insecurity is increasing particularly among low-income adults, and treatment for chronic diseases has become suboptimal in many LMICs [38]. However, our findings must be interpreted in light of several limitations. First, the study was cross-sectional in nature and thus, temporal associations could not be established although in the context of food insecurity and multimorbidity, the association is likely to be bidirectional. Second, most data were self-reported potentially introducing social desirability and recall bias into the findings. Third, our measure of food insecurity was based on two questions and did not constitute a comprehensive food insecurity measure. Finally, our list of chronic diseases included a variety of diseases which are highly prevalent in LMICs, but lacked some diseases, such as cancer which is associated with particularly high costs to treat. Thus, it is possible for the results to have changed with a different set of chronic conditions.

## Conclusion

In conclusion, food insecurity was significantly associated with higher odds for multimorbidity in our study on older adults from LMICs, although significant associations were not found in all countries. Future longitudinal studies that assess the temporal association, and experimental studies that examine the underlying mechanisms, as well as qualitative studies to identify the reasons for the between-country heterogeneity are warranted to inform targeted interventions.

## Supplementary Information

Below is the link to the electronic supplementary material.

Supplementary file1 (DOCX 17 KB)
