# Correction to: Food insecurity and physical multimorbidity among adults aged ≥ 50 years from six low- and middle-income countries

**DOI:** 10.1007/s00394-022-03082-9

**Published:** 2023-01-11

**Authors:** Lee Smith, Jae Il Shin, Louis Jacob, Guillermo F. López Sánchez, Felipe Schuch, Mark A. Tully, Hans Oh, Nicola Veronese, Pinar Soysal, Laurie Butler, Yvonne Barnett, Ai Koyanagi

**Affiliations:** 1 Centre for Health, Performance and Wellbeing, Anglia Ruskin University, Cambridge, UK; 2 Department of Pediatrics, Yonsei University College of Medicine, Seoul, 03372 Korea; 3 Research and Development Unit, Parc Sanitari Sant Joan de Déu, CIBERSAM, ISCIII, Dr. Antoni Pujadas, 42, Sant Boi de Llobregat, 08830 Barcelona, Barcelona Spain; 4 Faculty of Medicine, University of Versailles Saint-Quentin-en-Yvelines, 78000 Versailles, France; 5 Division of Preventive Medicine and Public Health, Department of Public Health Sciences, School of Medicine, University of Murcia, Murcia, Spain; 6 Department of Sports Methods and Techniques, Federal University of Santa Maria, Santa Maria, Brazil; 7 School of Health Sciences, Institute of Mental Health Sciences, Ulster University, Newtownabbey, BT15 1ED Northern Ireland; 8 Suzanne Dworak Peck School of Social Work, University of Southern California, Los Angeles, CA 90007 USA; 9 Department of Internal Medicine and Geriatrics, University of Palermo, 90133 Palermo, Italy; 10 Department of Geriatric Medicine, Bezmialem Vakif University, 34093 Istanbul, Turkey; 11 ICREA, Pg, Lluis Companys 23, 08010 Barcelona, Spain; 12 Faculty of Health Sciences, Universidad Autónoma de Chile, Providencia, Chile

**Correction to: European Journal of Nutrition** 10.1007/s00394-022-02999-5

The original version of this article unfortunately contained a mistake. The second affiliation for author Felipe Schuch was missing and second affiliation should be

Faculty of Health Sciences, Universidad Autónoma de Chile, Providencia, Chile.

